# TRIM5α associates with proteasomal subunits in cells while in complex with HIV-1 virions

**DOI:** 10.1186/1742-4690-8-93

**Published:** 2011-11-12

**Authors:** Zana Lukic, Stéphane Hausmann, Sarah Sebastian, Justin Rucci, Jaya Sastri, Seth L Robia, Jeremy Luban, Edward M Campbell

**Affiliations:** 1 Department of Microbiology and Immunology, Stritch School of Medicine, Loyola University Chicago, Maywood IL, USA; 2 Department of Microbiology and Molecular Medicine, University of Geneva, Geneva, Switzerland; 3 Department of Cell and Molecular Physiology, Stritch School of Medicine, Loyola University Chicago, Maywood IL, USA

**Keywords:** TRIM5α, HIV-1, proteasomal subunits, cytoplasmic bodies, immunofluorescence

## Abstract

**Background:**

The TRIM5 proteins are cellular restriction factors that prevent retroviral infection in a species-specific manner. Multiple experiments indicate that restriction activity requires accessory host factors, including E2-enzymes. To better understand the mechanism of restriction, we conducted yeast-two hybrid screens to identify proteins that bind to two TRIM5 orthologues.

**Results:**

The only cDNAs that scored on repeat testing with both TRIM5 orthologues were the proteasome subunit PSMC2 and ubiquitin. Using co-immunoprecipitation assays, we demonstrated an interaction between TRIM5α and PSMC2, as well as numerous other proteasome subunits. Fluorescence microscopy revealed co-localization of proteasomes and TRIM5α cytoplasmic bodies. Forster resonance energy transfer (FRET) analysis indicated that the interaction between TRIM5 and PSMC2 was direct. Previous imaging experiments demonstrated that, when cells are challenged with fluorescently-labeled HIV-1 virions, restrictive TRIM5α orthologues assemble cytoplasmic bodies around incoming virion particles. Following virus challenge, we observed localization of proteasome subunits to rhTRIM5α cytoplasmic bodies that contained fluorescently labeled HIV-1 virions.

**Conclusions:**

Taken together, the results presented here suggest that localization of the proteasome to TRIM5α cytoplasmic bodies makes an important contribution to TRIM5α-mediated restriction.

## Background

The species-specific tropism of numerous retroviruses is determined by host cell proteins, termed restriction factors, which inhibit viral replication at various stages of the viral life cycle. Many members of the TRIM family of proteins act as viral restriction factors. One well-characterized example is the ability of TRIM5α from rhesus macaques (rhTRIM5α) to inhibit human immunodeficiency virus type-1 (HIV-1) [1,2]. TRIM5α contains RING, B-box, coiled-coil, and B30.2/SPRY domains [3]. The RING domain of TRIM5α has E3-ubiquitin ligase activity, which is important for restriction and self-ubiquitination. When certain residues within the RING domain are mutated, TRIM5α loses the ability to potently restrict HIV-1 and self-ubiquitinate, demonstrating the role of ubiquitination during TRIM5α restriction [1]. It is well established that the restriction requires an interaction between the viral capsid lattice and the B30.2/SPRY domain of TRIM5α [4-6]. Following the binding of the viral core, TRIM5α mediates an event or series of events that result in the abortive disassembly of the viral core in a manner that prevents the accumulation of reverse transcription (RT) products [2,7,8]. Proteasome inhibitors prevent TRIM5α mediated inhibition of RT products [8,9] and abortive disassembly of the viral core [10,11] without affecting the ability of TRIM5α to inhibit retroviral infection [8,9]. Additionally, TRIM5α itself is degraded in a proteasome dependent fashion following cytoplasmic delivery of restriction sensitive virus [12].

However, these studies relied on pharmacological inhibitors of proteasome function, which can have pleiotropic effects on the biology of cells. Specifically, proteasome inhibitors such as MG132 deplete the cellular pool of ubiquitin available for cellular processes distinct from degradation. For example, it was recently shown that TRIM5α can mediate the formation of unanchored K63-linked polyubiquitin chains during restriction, which require free cellular ubiquitin and are thought to activate signaling pathways in a manner independent of proteasome activity [13]. In studies relying on proteasomal inhibitors, it is difficult to discriminate between a direct role for proteasomal degradation or an indirect depletion of free cellular ubiquitin that perturbs the generation of K63-linked polyubiquitin chains [13].

Here, we provide evidence for a direct connection between TRIM5α and the proteasome machinery. The 26S proteasome is a barrel shaped, multiprotein complex consisting of a 20S core particle (CP) and 19S regulatory particle (RP) [14]. The 20S CP is composed of heteroheptameric rings, two outer α-rings and two inner β-rings, each consisting of seven structurally similar α and β subunits. The 19S RP contains a base, consisting of RPT and RPN subunits and a regulatory lid comprised of RPN subunits [14]. These subunits were named in studies initially performed using yeast derived proteins, and the vast majority of these names have been maintained in the nomenclature describing mammalian proteasomes. One relevant exception important in this study is the RPT1 subunit, generally referred to as PSMC2. In this study, we use the term PSMC2, though many studies utilize the term RPT1 to describe the mammalian homologue of this protein [14-16]. We observe an interaction between TRIM5α and the proteasomal subunit PSMC2 using yeast-two-hybrid analysis. This interaction is confirmed using co-immunoprecipitation assays and deconvolution microscopy showing subunits of the proteasome localizing to TRIM5α cytoplasmic bodies. Using Forster Resonance Energy Transfer (FRET) analysis, we show that TRIM5α closely associates (> 5 nm separation) with proteasomal subunits in cytoplasmic bodies. Finally, we detect the presence of proteasomal subunits in TRIM5α cytoplasmic bodies associated with restriction sensitive virions.

## Results

### TRIM5α associates with PSMC2 and ubiquitin in a yeast-two hybrid system

**Figure 1:**
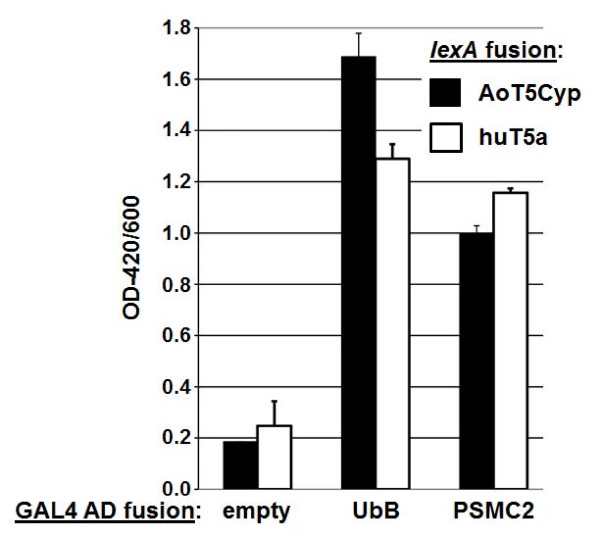
**Ubiquitin and PSMC2 identified as interacting proteins with huTRIM5α and TRIMCyp via yeast two-hybrid**. Yeast strain bearing pSH18-34 was transformed with the bait plasmid pEG202-human TRIM5α or the bait plasmid pEG202-TRIMCyp. Sequentially, a pool of pJG4-5 prey plasmids containing a cDNA library were transfected, and screening for positive interaction proteins was performed. Screening identified two GAL4 AD fusions that activated gene transcription by lexA fusions to human and Aotus TRIM5α proteins that were sequenced and submitted for BLAST analysis.

To identify binding proteins of TRIM5, we performed yeast two-hybrid screens using human TRIM5α and Aotus owl monkey TRIM5Cyp fused to lexA as baits. These baits were screened against a human cDNA library fused to prey. This yielded approximately 3 × 10^6 ^transformants for screening. Screening for interaction-positive colonies was performed on selective medium in order to induce the expression of cDNAs in the library. Subsequently, colonies were tested for β-galactosidase expression and positive clones were subjected to DNA sequencing and BLAST analysis. From this data, proteasomal subunit PSMC2 and ubiquitin were identified as interacting proteins of human TRIM5α. These hits were individually verified by repeating the two-hybrid system using individual PSMC2 and ubiquitin clones. These clones were found to interact with huTRIM5α and aotus TRIM5Cyp (Figure 1).

### TRIM5α associates with PSMC2 and other proteasomal subunits by co-immunoprecipitation

**Figure 2:**
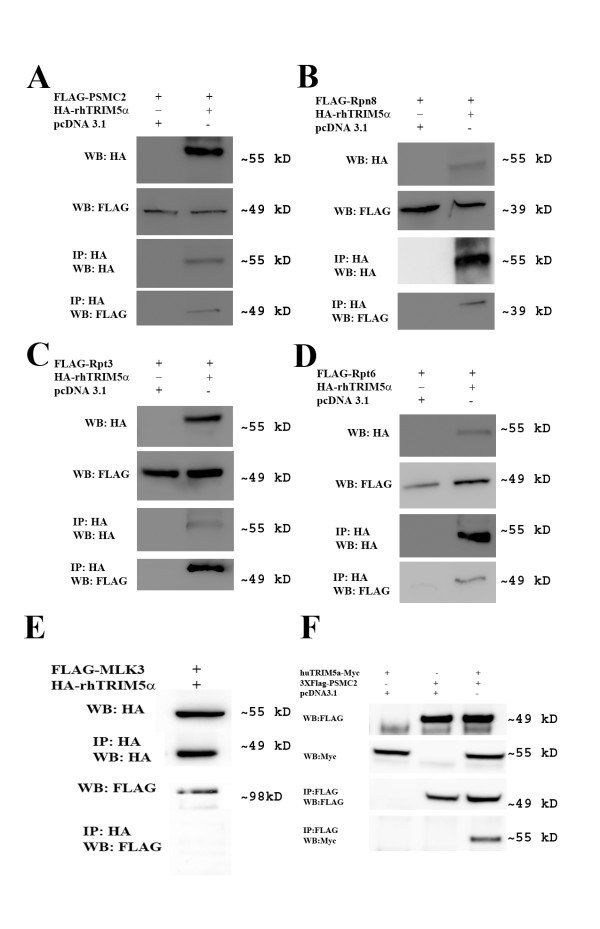
**TRIM5α specifically co-immunoprecipitates with proteasome subunits**. (A) Co-immunoprecipitation of PSMC2 with rhTRIM5α. 293T cells were transfected with HA-rhTRIM5α and FLAG-PSMC2 or pcDNA3.1 empty vector and FLAG-PSMC2 and subjected to immunoprecipitation (IP) with an HA antibody followed by Western blot using indicated antibodies. (B) Co-immunoprecipitation of RPN8 with rhTRIM5α. 293T cells were transfected with HA-rhTRIM5α and FLAG-RPN8 or pcDNA3.1 empty vector and FLAG-RPN8 and subjected to immunoprecipitation (IP) with an HA antibody followed by Western blot using indicated antibodies. (C) Co-immunoprecipitation of RPT3 with rhTRIM5α. 293T cells were transfected with HA-rhTRIM5α and FLAG-RPT3 or pcDNA3.1 empty vector and FLAG-RPT3 and subjected to immunoprecipitation (IP) with an HA antibody followed by Western blot using indicated antibodies. (D) Co-immunoprecipitation of RPT6 with rhTRIM5α. 293T cells were transfected with HA-rhTRIM5α and FLAG-RPT6 or pcDNA3.1 empty vector and FLAG-RPT6 and subjected to immunoprecipitation (IP) with an HA antibody followed by Western blot using indicated antibodies. (E) 293T cells were transfected with HA-MLK-3 and subjected to immunoprecipitation with an HA antibody followed by Western Blot. (F) Co-immunoprecipitation of PSMC2 with huTRIM5α. 293T cells were transfected with Myc-rhTRIM5α and FLAG-PSMC2 or pcDNA3.1 empty vector and FLAG-PSMC2 and subjected to immunoprecipitation (IP) with a FLAG antibody followed by Western blot using indicated antibodies.

To examine the association between TRIM5α proteins and proteasomal subunits in human cells, 293T cells were transfected with HA-rhTRIM5α and FLAG-PSMC2. Following pulldown with anti-HA antibody and blotting with anti-FLAG antibody, we detected FLAG-PSMC2 when HA-rhTRIM5α was present, but not when pcDNA3.1 vector control was present (Figure 2A), demonstrating an association between PSMC2 and rhTRIM5α. To determine if this association was specific to PSMC2 or other subunits of the proteasome, we, similarly, transfected HA-rhTRIM5α with FLAG-tagged versions of the proteasomal subunits RPT3, RPT6, and RPN8 (Figure 2B, C, D). All three subunits were specifically pulled down with HA-rhTRIM5α, indicating that rhTRIM5α associates with numerous subunits of the 26S proteasome. To ensure the specificity of this pulldown, we performed a similar pulldown with FLAG-tagged Mixed Lineage Kinase 3 (MLK3) [17]. FLAG-MLK3 did not co-immunoprecipitate with HA-rhTRIM5α (Figure 2E). This demonstrates the specificity of this interaction detected by our immunoprecipitation protocol. Additionally, huTRIM5α-Myc was immunoprecipitated with FLAG-PSMC2 following transfection in HEK293 cells (Figure 2F), establishing that the association of proteasomal subunits with TRIM5α is conserved across species.

### Proteasomal subunits localize to rhTRIM5α assemblies in cells

**Figure 3:**
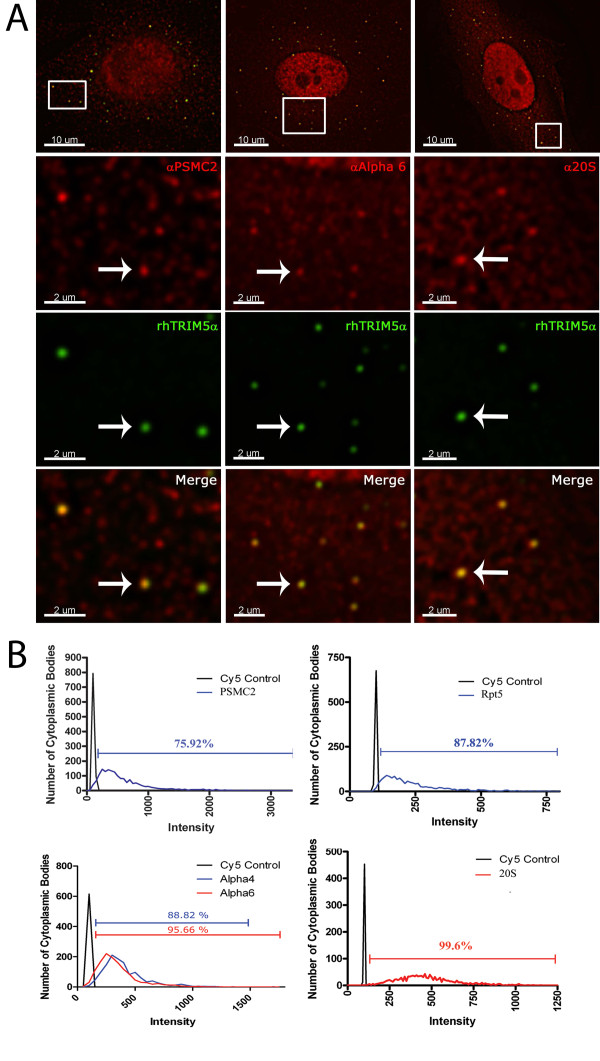
**Proteasomal subunits localize to TRIM5α assemblies at various frequencies**. (A) HeLa cells stably expressing YFP-rhTRIM5α were fixed and immunostained with antibodies specific to the individual subunits of the proteasome. Z-stack images were collected with a DeltaVision microscope equipped with a digital camera using a 1.4-numerical aperture (NA) 100× objective lens, and were deconvolved with SoftWoRx deconvolution software. Individual channel images were superimposed to create the merged panels. (B) Deconvolved images were analyzed for subunit mean fluorescence intensity (MFI) in YFP-rhTRIM5α cytoplasmic bodies by the use of the Surface Finder function in the Imaris software (Bitplane). For each YFP-rhTRIM5α cytoplasmic body, the MFI of the subunit was determined and the data was plotted in GraphPad Prism 5^® ^software.

Next we sought to examine the localization of proteasomal subunits in HeLa cells stably expressing YFP-rhTRIM5α [18]. Previously, we were unable to detect the localization of rhTRIM5α and the 20S proteasome using a polyclonal antibody [11]. However, the results described above prompted us to speculate that this antibody did not accurately represent the localization of proteasomal subunits by immunofluorescence. We, therefore, initiated a more comprehensive study of proteasome localization using a large panel of antibodies to subunits of the proteasome. As shown in Table 1 and additional file 1, these antibodies typically fell into two categories when utilized for immunofluorescence: those in which a pronounced nuclear localization of the specified subunit was observed and those in which a pronounced nuclear localization was not observed. Numerous reports have shown that proteasome subunits, in addition to maintaining a noticeable and biologically relevant cytoplasmic fraction, exhibit a pronounced nuclear localization [19]. Some antibodies in the panel examined as well as the antibody used in the previous study did not exhibit pronounced nuclear staining (Table 1), casting doubt on the utility of these antibodies for detecting proteasomal subunits by immunofluorescence. In contrast, the majority of antibodies did reveal a pronounced nuclear staining by immunofluorescence. Therefore, we used these antibodies to determine if proteasomal subunits localize to rhTRIM5α assemblies in HeLa stable cell lines expressing YFP-rhTRIM5α [18]. Antibodies to numerous subunits demonstrated pronounced accumulation of proteasomal subunits in these assemblies (Figure 3). Specifically, PSMC2 could be detected in these assemblies (Figure 3A). Antibodies to the proteasomal subunits α2, α4, α6, and RPT5 also detected a pronounced accumulation of these proteins in YFP-rhTRIM5α assemblies. We also detected proteasomal subunits associated with YFP-rhTRIM5α cytoplasmic assemblies using a rabbit polyclonal antibody to the 20S core particle of the proteasome (Figure 3A). To determine if proteasomal subunit localization was ubiquitous or if localization was specific to a subset of rhTRIM5α cytoplasmic assemblies, we quantified the proteasome specific immunofluorescent signal associated with individual cytoplasmic assemblies identified by automated image analysis. This analysis revealed that the vast majority of YFP-rhTRIM5α assemblies contained proteasomal subunits. Using an antibody to the 20S core, 99.6% of subunits had staining levels above background, which was defined as the staining observed using secondary antibodies in the absence of primary antibodies (Figure 3B). Similar results were also observed using mouse monoclonal antibodies to PSMC2 and another base subunit RPT5 as well as α4 and α6 subunits (Figure 3B). Because of the strong degree of 20S core staining observed in YFP-rhTRIM5α assemblies using a rabbit polyclonal antibody, this suggests that virtually all TRIM5α assemblies associate with proteasomes. Therefore, we believe that alterations in the percentage of cytoplasmic bodies containing individual proteasomal subunits we observed using antibodies represent the ability of these antibodies to reliably detect subunits that are likely present in these assemblies. However, we cannot exclude the possibility that individual subunits are present in more or less abundance, as proteasomal subunit populations may conditionally vary [20,21]

**Table 1:** Characterization of proteasome antibodies for immunofluorescence.

Proteasome Antibodies			
**19S Base**	**Catalog Number**	**Ab Nuclear Localization**	**Locialization with rhTRIM5a**

RPT1 mouse	BML-PW8825	INTERMEDIATE	YES

RPT1 rabbit	BML-PW8165	INTERMEDIATE	YES

RPT2 rabbit	BML-PW8305	NO	NO

RPT3 mouse	BML-PW8220	YES	NO

RPT3 rabbit	BML-PW8175	INTERMEDIATE	YES

RPT4 mouse	BML-PW8310	INTERMEDIATE	NO

RPT4 rabbit	BML-PW8830	NO	NO

RPT5 mouse	BML-PW8770	YES	YES

RPT5 rabbit	BML-PW8765	NO	NO

RPT6 rabbit	BML-PW8320	YES	NO

**20S Alpha**			

2 mouse	BML-PW8105	YES	YES

4 mouse	BML-PW8120	YES	YES

5 mouse	BML-PW8125	NO	NO

6 mouse	BML-PW8100	YES	YES

7 mouse	BML-PW8110	INTERMEDIATE	NO

Core rabbit	BML-PW8155	YES	YES

Alpha 1,2,3,5,6 & 7 mouse	BML-PW8195	NO	NO

20S Beta			

1i (LMP2-13) rabbit	BML-PW8345	NO	NO

1 mouse	BML-PW8140	NO	NO

2 mouse	BML-PW8145	NO	NO

2i (MECL-1) rabbit	BML-PW8350	YES	NO

3 mouse	BML-PW8130	INTERMEDIATE	NO

4 rabbit	BML-PW8890	NO	NO

5i (LMP7) mouse	BML-PW8845	NO	NO

5i (LMP7) rabbit	BML-PW8355	YES	NO

5 rabbit	BML-PW8895	NO	NO

6 rabbit	BML-PW9000	NO	NO

7 mouse	BML-PW8135	NO	NO

Antibodies were characterized based on their localization in the nucleus. Previous reports have shown that a large pool of proteasomes are located in the nucleus while a fraction remains in the cytoplasm thus the nuclear staining represents antibodies deemed successful for immunofluorescence.

### FRET analysis reveals a direct association between rhTRIM5α and proteasomal subunits

**Figure 4:**
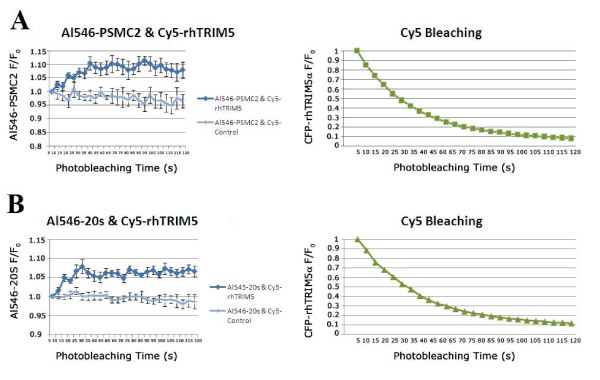
**TRIM5α associates with PSMC2 and 20S**. (A) Cells stably expressing HA-rhTRIM5α were seeded on glass coveslips and immunostained for HA using Cy5 rabbit antibody and endogenous proteasome subunit PSMC2 using Al546 mouse antibody. Following staining procedure, cells were fixed and mounted on glass slides. Cy5 channel was progressively bleached and fluorescence intensity was detected over time in Al546 and Cy5 channels. Images were analyzed using softWoRx software. (B) Cells stably expressing HA-rhTRIM5α were immunostained for HA using Cy5 rabbit antibody and endogenous 20S core particle using Al546 mouse antibody. Cy5 channel was progressively bleached and fluorescence intensity was detected over time in Al546 and Cy5 channels. Images were analyzed using softWoRx software.

To better understand the association between rhTRIM5α and proteasomal subunits, we performed immunofluorescence based Forster Resonance Energy Transfer (FRET) analysis on immunofluorescently labeled rhTRIM5α assemblies and proteasomal subunits. For these studies, we utilized a well-characterized HeLa cell line stably expressing HA-rhTRIM5α [2]. As previously observed with our YFP-rhTRIM5α cell line, proteasomal subunits could be observed to localize to rhTRIM5α assemblies in these cells (Additional File 2). We labeled HA-rhTRIM5α and proteasomal subunits using secondary antibody combinations that have been previously used to measure FRET interactions in cells [22]. To measure FRET, we utilized the acceptor photobleaching approach, in which the acceptor of a FRET pair is serially photobleached, and the fluorescence of the donor fluorophore is measured over time [23-25]. In this system, if FRET occurs between two fluorophores, then bleaching of the acceptor will result in an increase in the fluorescence of the donor fluorophore as the acceptor fluorophore becomes unable to absorb the energy released from the donor. When PSMC2 was labeled using Alexa546 (donor) and HA-rhTRIM5α was labeled using Cy5 (acceptor), serial photobleaching of Cy5 resulted in an increase in the fluorescence detected for PSMC2 (Alexa546). Control bleaching of Cy5 in cells stained with Cy5 secondary antibody alone, in the absence of αHA primary antibody, did not exhibit this pattern (Figure 4A). Similar results were obtained when a rabbit polyclonal antibody to the 20s proteasome was used (Figure 4B). FRET measurement is generally accepted to indicate a direct (< 5 nm) association between two proteins. In this case, because both primary and secondary antibodies were used in this assay, we cannot definitively state this to be true because the addition of a secondary antibody, which has a hydrodynamic radius of 5.5 nm [26] results in a two-fold decrease in the resolution of this assay (> 10 nm). However, these experiments provide evidence that TRIM5α and proteasomal subunits exist in very close proximity in rhTRIM5α assemblies, well below what can be observed using colocalization analysis (Figure 3), which is limited by the resolution limit of light microscopy (~200 nm).

**Figure 5:**
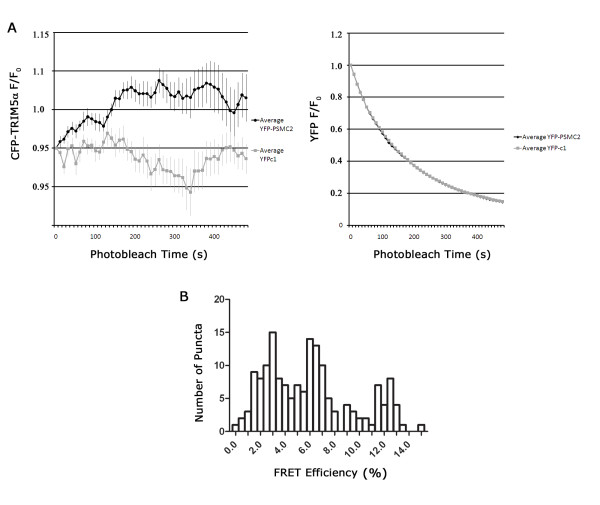
**TRIM5α directly associates with proteasome subunit PSMC2**. (A) Progressive acceptor photobleaching of YFP-PSMC2 resulted in an increase in CFP-rhTRIM5α fluorescence indicating FRET. YFP-PSMC2 or YFP empty vector and CFP-rhTRIM5α were transfected in HeLa cells at a 1:1 ratio. Over the course of 10 minutes, 50 frames were obtained for YFP and CFP channels. (B) Fluorescence intensities of CFP-rhTRIM5α and YFP-PSMC2 are recorded. EFRET is calculated as a relative increase of YFP fluorescence intensity (%) after photobleaching of the CFP FRET acceptor.

To determine if these proteins were within proximity (typically less than 5 nm) and therefore directly interacting, we performed FRET analysis using fusion proteins in which rhTRIM5α and PSMC2 were fused to the commonly used FRET pair of CFP and YFP, respectively. In live cells transfected with both CFP-rhTRIM5α and YFP-PSMC2, colocalization of rhTRIM5α and PSMC2 could be readily observed (Additional File 3). We then utilized acceptor photobleaching to determine if FRET occurred between these two proteins in areas of notable colocalization. In this method, the acceptor (YFP-PSMC2) is serially photobleached. FRET is subsequently measured as an increase in donor fluorescence (CFP-rhTRIM5α) which occurs as the acceptor is bleached and therefore no longer absorbs the resonant energy from the donor (Additional File 3) [27]. In these experiments, notable increases in the CFP-rhTRIM5α fluorescence were observed following YFP-PSMC2 photobleaching but an increase was not detected when YFP empty vector was photobleached (Figure 5A). While the FRET difference between YFP-PSMC2 and YFP empty vector samples was statistically significant (p < 0.0001), there were also numerous CFP-rhTRIM5α assemblies in which photobleaching of YFP-PSMC2 did not induce an apparent increase in CFP-rhTRIM5α fluorescence (data not shown). This suggests that these two proteins do not directly interact in some assemblies. Although we attempted to focus our analysis only on assemblies that did not enter or leave the plane of focus during the course of the experiment, we were concerned that the disparate results obtained were a result of the movement of some assemblies relative to the focal plane during the analysis period. To address this concern, we also performed E-FRET analysis on these assemblies by measuring the amount of YFP fluorescence induced following CFP excitation. Because this method allows a calculation of FRET efficiency derived from three individual images taken in rapid succession, movement of TRIM5α assemblies during the acquisition period was not a concern. When this method of analysis was utilized, we again observed that the FRET efficiencies of individual assemblies varied considerably (Figure 5B).

It is somewhat surprising that our immunofluorescence based FRET assay consistently exhibited FRET while our fusion protein based assay indicated significant variability in the degree of FRET observed. However, both methods collectively demonstrate that, in some assemblies, a direct interaction between rhTRIM5α and PSMC2 exists.

### Proteasomal subunits are present in rhTRIM5α assemblies containing HIV-1 virions

**Figure 6:**
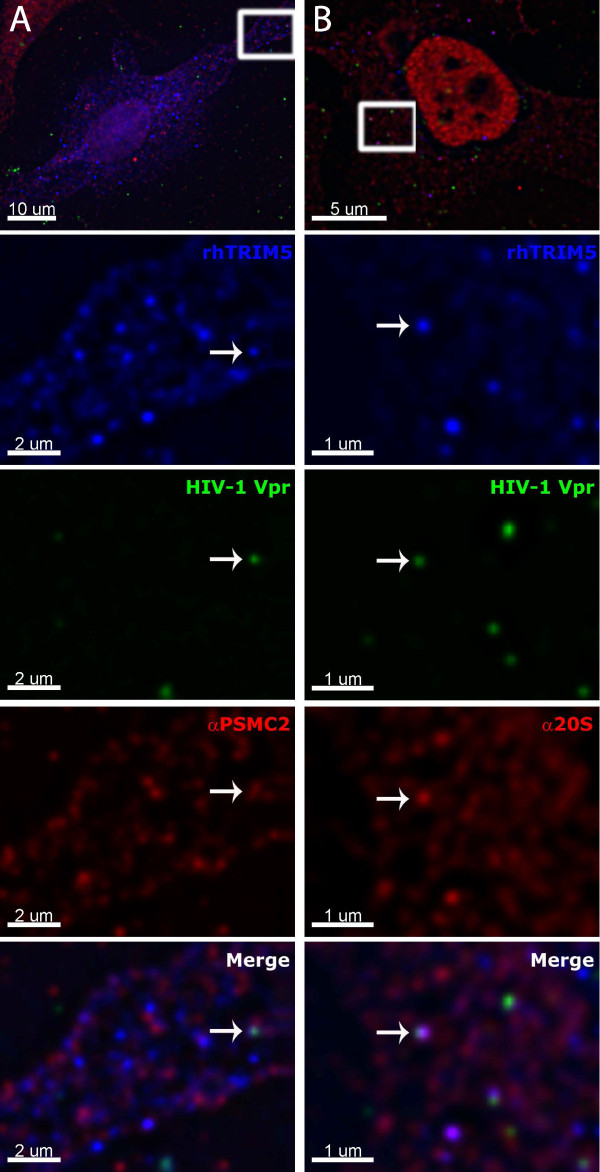
**Proteasome associates with TRIM5α cytoplasmic bodies containing restriction sensitive virus**. HeLa cells stably expressing YFP-rhTRIM5α at low levels were seeded on glass coverslips and infected with VSVg pseudotyped HIV-1 virions containing fluorescently tagged mCherry-Vpr for 30 minutes at 37°. Following infection, cells were fixed and stained for the endogenous (A) PSMC2 proteasomal subunits and (B) 20S core. Z-stack images were collected and deconvolved using Deltavision deconvolution software. Individual channel images are superimposed to create the merged panels.

The studies above examined the localization of proteasomal subunits to rhTRIM5α assemblies that exist in cells in the absence of virus. The degree to which pre-existing rhTRIM5α cytoplasmic bodies resemble assemblies that form around individual virions [11] is unclear. One recent study has found that TRIM5α forms hexagonal protein assemblies in the presence or absence of *in vitro *assembled hexameric capsid structures [28], suggesting that TRIM5α forms structurally similar assemblies in the presence or absence of restriction sensitive virus. The tendency to form such assemblies is enhanced by the presence of these hexameric capsid assemblies [29]. However, there may be biologically important differences between cytoplasmic assemblies of TRIM5α that form around a restriction sensitive virus and those that form in the absence of virus. We, therefore, sought to determine if TRIM5α assemblies that form around restriction sensitive virus also contain proteasomal subunits. To this end, we infected a HeLa cell line stably expressing YFP-rhTRIM5α with low levels of pre-existing cytoplasmic bodies with VSV-g pseduotyped HIV-1 virions fluorescently labeled with an mCherry-Vpr fusion protein [11,30]. Following infection for 30 minutes, cells were fixed, stained for proteasomal subunits and quantified for any colocalization between rhTRIM5α formed cytoplasmic bodies, restriction sensitive virus, and proteasomal subunits (Figure 6). As the engagement of the viral capsid by rhTRIM5α rapidly leads to the loss of virally associated fluorescent signal, [11], only a small percentage of viral particles could be observed associating with TRIM5α in fixed cell images in the absence of proteasome inhibitor, as we have previously reported [11]. However, when we did detect such complexes, both proteasomal subunit PSMC2 and the 20S core localized to cytoplasmic assemblies of TRIM5α that formed around a restriction sensitive virus. When these images were quantified, approximately 60% of TRIM5α cytoplasmic bodies that contained virus also contained the 20S proteasome (data not shown), demonstrating that proteasomal subunits are recruited to TRIM5α assemblies which associate with HIV-1 viral complexes.

## Discussion

Previous studies utilizing pharmacological proteasome inhibitors have suggested a role for proteasome mediated degradation in rhTRIM5α restriction [8,9,11,12]. However, evidence demonstrating an actual interaction between rhTRIM5α and the proteasome has not been published. Due to the potential pleiotropic effects of proteasomal inhibitors on cellular function, such as depletion of the free cellular ubiquitin, it is possible that previously obtained results may not represent a direct role for the proteasome in HIV-1 restriction mediated by TRIM5α. Indeed, recent data have described a proteasome independent role for ubiquitin and rhTRIM5α E3- ligase activity in retroviral restriction [13]. In this work, we utilized four methods to demonstrate an interaction between proteasomal subunits and rhTRIM5α. Yeast two-hybrid analysis of a cDNA library containing 9.6 × 10^6 ^clones identified PSMC2 as a huTRIM5α and TRIM5Cyp interacting protein. We also used co-immunoprecipitation and immunofluorescence localization studies to show an association between various proteasomal subunits, including PSMC2 and rhTRIM5α. Using FRET analysis, we show a direct interaction between PSMC2 and rhTRIM5α in cytoplasmic rhTRIM5α assemblies.

Notably, the majority of studies examining the turnover of TRIM5α have observed only a mild effect of proteasome inhibitors on TRIM5α turnover [8,10,31,32] suggesting that the turnover of TRIM5α in the absence of the virus occurs in a manner that is predominantly proteasome independent. We also observe that the cell lines used in these studies show similar sensitivity to proteasome inhibitors (data not shown). However, one study showed that, following exposure to restriction sensitive virus, TRIM5α degradation transitions to a proteasome dependent mechanism [12]. However, the mechanism by which TRIM5α becomes sensitive to proteasomal degradation remains unclear. Our FRET experiments suggest a possible explanation for this observation.

FRET analysis of the interaction between PSMC2 and rhTRIM5α provides insight into the nature of this association and its underlying biology. We observed an apparent discord between our immunofluorescence based FRET analysis and our fluorescent protein based FRET analysis. There are two possibilities to explain this discrepancy. First, our fluorescent protein based FRET may have yielded variable results because FRET measurements in this assay can be affected if the fluorescent fusion protein does not exhibit the same full biological activity as the native protein. While our previous studies have demonstrated that fluorescent tags do not alter the activity of rhTRIM5α restriction, it was not feasible to similarly characterize the biological activity of our YFP-PSMC2 construct, which may have contributed to some of the variability observed in these experiments. However, it is also possible that the differences between these assays were due to decreased spatial stringency afforded by the utilization of antibodies used to detect rhTRIM5α and endogenous PSMC2 in our immunofluorescent FRET. This decreased stringency may be due to: 1) the hydrodynamic radius of the antibody versus the fluorescent protein size or 2) a single donor can transfer energy to multiple acceptors in immunofluorescent FRET while the same phenomenon does not occur in the fluorescent protein based FRET. A single antibody has a hydrodynamic radius of 5.5 nm [33] while a fluorescent protein is 2-3 nm in size [34]. Therefore, the use of antibodies may have allowed FRET to occur despite the lack of direct interaction between PSMC2 and rhTRIM5α in all cases. A possible explanation for the observed heterogeneity using fluorescent protein based FRET is that another protein frequently separates rhTRIM5α from the proteasome machinery. In our previous work, we showed that p62 directly associates with rhTRIM5α using fluorescent protein FRET and that p62 siRNA knockdown increases the turnover rate of rhTRIM5α, reducing the steady state expression of TRIM5α [25]. We, therefore, speculate that the differences noted in our FRET analyses indicate that p62 may reduce the accessibility of rhTRIM5α to degradation in the absence of virus. In some assemblies, this sequestration of rhTRIM5α by p62 may be less efficient, allowing the proteasome machinery to engage rhTRIM5α in these assemblies. This would be consistent with the mild but incomplete sensitivity of TRIM5α turnover to proteasomal inhibitors [8,10,31]

We also show that proteasomal subunits associate with rhTRIM5α assemblies containing fluorescently labeled HIV-1 virions. We have previously shown that the formation of rhTRIM5α assemblies around HIV-1 virions leads to the rapid loss of virion fluorescent signal [11]. Taken together with our imaging data presented here, showing proteasomal subunits are present in rhTRIM5α/HIV-1 complexes, the data suggest that proteasomal subunits are rapidly recruited to these complexes and they are important to the restriction process mediated by TRIM5α. Mechanistically how the proteasome is engaged during the restriction process is not known. We detected proteasomal subunits in a greater percentage of preexisting cytoplasmic bodies (> 90%) (Figure 3) compared to cytoplasmic bodies containing HIV-1 virions (~60%). The simplest explanation for this observation is that proteasomal subunits are recruited to cytoplasmic bodies which form *de novo *around a restriction sensitive viral core. This would be consistent with other studies which have found that preexisting cytoplasmic bodies are not required for restriction [35,36]. The recruitment of proteasomal subunits to this complex, sufficient to be detected by immunofluorescence, would then occur. Our fixed cell imaging likely detected complexes in various stages of this process, leading to a reduced number of these assemblies containing detectable amount of proteasomal subunits, compared to preexisting cytoplasmic bodies.

Precisely how the proteasome is involved in restriction is unclear. It is possible that the proteasome recognizes the virus complexed with TRIM5α, leading to direct degradation of viral components. An alternative possibility is that that the proteasome recognizes TRIM5α directly, which is itself degraded in a manner that leads to the abortive disassembly of the viral core without the direct degradation of viral proteins. The foundation for either possibility has been described here, in which we show a direct role for the proteasome in rhTRIM5α-mediated restriction. In the future, we will pursue experiments to explore either possibility.

## Conclusions

Here, we show that proteasomal subunits associate with TRIM5α in multiple ways, and this association also occurs while in complex with restriction sensitive HIV-1 virions. These findings suggest that association of the proteasome with TRIM5α is relevant to TRIM5 biology and restriction.

## Materials and methods

### Yeast Two Hybrid Screen

The yeast-two-hybrid screen was based on previously described reagents and protocols [37]. Yeast strain EGY48 (*MAT*a *trp1 his3 LEU2*::pLexAop6-*LEU2*) bearing pSH18-34, a reporter plasmid containing 8 lexA-binding sites that direct transcription of *lacZ*, was sequentially transformed with the bait plasmid pEG202-humanTRIM5α, or the bait plasmid pEG202-TRIMCyp. Then yeast was transformed with pJG4-5 (prey), > 90% of which contain a HeLa cell cDNA library, in a pool of 9.6 × 10^6 ^individual clones. After each transformation, colonies were selected on synthetic drop-out medium. Transformation efficiency for the prey plasmid was 10^5 ^colonies per μg DNA, and 25 μg of the library plasmid pool were used to achieve a total of approximately 2-3 × 10^6 ^colonies for screening. Screening for interaction-positive colonies was performed on selective, galactose-containing medium in order to induce the expression of the cDNAs in the library. Subsequently, a filter-lift assay was used to confirm positive clones. Briefly, filters were used to lift colonies from media plates, placed at -80°C for 15 minutes to disrupt yeast cell walls, and incubated on Whatman paper soaked in buffer Z containing X-gal. Colony lifts that turned blue within 30 minutes at 37°C were termed putative positive interactors, and the corresponding colonies were picked and analyzed further. Library-cDNA containing plasmids (pJG4-5) were recovered from positive clones. Since these clones contained all three plasmids (reporter, bait, and prey), it was necessary to make use of the KC8 *E. coli *strain to isolate pJG4-5. KC8 bacteria are *trp- *and can be selected on TRP- plates that only allow growth if the bacteria contain pJG4-5 (TRP153 selection marker). The library inserts of recovered pJG4-5 plasmids were sequenced, submitted to BLAST analysis, and retested for activity in the two-hybrid system as individual clones.

### Recombinant DNA Constructs

HA-tagged rhTRIM5α and huTRIM5α-Myc have been previously described [38,39]. FLAG-tagged proteasomal subunits were generously provided by Dr. Shigeo Murata [40]. 3X-FLAG PSMC2 was generated by PCR-amplification of the PSMC2 open reading frame from pCMV6-AC-PSMC2 plasmid (Origene) using primers that introduced unique *Spe*I and *Not*I sites at the start codon and 3' of the stop codon, respectively. The PCR product was digested with *Spe*I and *Not*I and then inserted into a customized pcDNA3.1 (-)-3XFlag expression vector. This generated PSMC2 was fused to an N-terminal 3X-FLAG. The insert of the resulting pcDNA3.1 (-) 3XF-PSMC2 plasmid was sequenced completely to confirm the presence of the wild-type open reading frame and exclude the acquisition of unwanted mutations during amplification. Yellow fluorescent protein (YFP) C1-PSMC2 was generated by PCR amplifying PSMC2 from a FLAG-PSMC2 plasmid that was generously provided by Dr. Shigeo Murata using the following forward primer 5'-GCGCGAAGCTTGGATGCCGGATT-3' and reverse primer 5'-GCGCGTCGACTCATCAGTTGTATG-3'. The PCR product was inserted into YFP C1 vector (Clontech) using *Hind*III and *Sal*I (New England Biolabs) restriction sites. The YFP C1-PSMC2 clone was confirmed with restriction digest and sequencing analysis to confirm the presence of the wild type open reading frame and fluorescent protein.

### Cell culture, transfection and virus production

HeLa and 293T cells were cultured in complete DMEM containing 10% fetal bovine serum, penicillin (final concentration 100 U/ml), and streptomycin (final concentration 100 μg/ml). mCherry-Vpr labeled virus was produced by Polyethylenimine (PEI) transfection [41] of 293T cells with 3.3 μg of pVSV-G, 5 μg of the proviral construct R7Δ EnvGFP in which the Nef gene was replaced with GFP and 1.7 ug mCherry-Vpr. Virus was harvested as previously described [42].

### Stable cell lines

HeLa cells stably expressing HA-rhTRIM5α [43] or YFP-rhTRIM5α [18] have been previously described. To generate HeLa cells to measure cytoplasmic body formation during viral infection, cells were transduced with YFP-rhTRIM5α retroviral vector and then selected in G418 (400 μg/mL) containing media. Single colony clones were screened by immunofluorescence to identify a cell line that expressed YFP-rhTRIM5α containing reduced pre-existing cytoplasmic bodies in the absence of restriction sensitive virus.

### Antibodies

Antibodies to various proteasomal subunits were purchased from Enzo Life Sciences (Table 1) and rabbit anti-PSMC2 was purchased from Santa-Cruz. Monoclonal Anti-FLAG M2-peroxidase antibody was purchased from Sigma (Catalog Number: A8592), and Anti-HA-peroxidase antibody was purchased from Roche (Catalog Number: 12013819001).

### Co-Immunoprecipitation

#### Rhesus TRIM5α co-immunoprecipitation

Sub-confluent 293T cells grown in 10-cm dishes were transfected with 10 ug of total plasmid DNA using PEI. 48-hours post transfection cells were washed with 1 ml ice-cold phosphate buffered saline (PBS) and lysed with 1 ml ice-cold lysis buffer (50 mM Tris, pH 7.4, 125 mM NaCl, 1% NP-40) supplemented with phosphatase inhibitor cocktail (Roche). Crude cell lysates were collected, transferred to a pre-chilled 2 ml microcentrifuge tube, and solubilized at 4° for one hour. Following solubilization, cells were sonicated for 10 seconds and centrifuged at 13,000- × *g *for 20 minutes at 4°C. 50 uL of supernatant were aliquoted for total cell lysate, and an equal volume of 2X Laemmli sample buffer was added; samples were then boiled for five minutes at 100°C. To pull down HA-rhTRIM5α, anti-HA antibody (Sigma) was added to the remaining supernatant at a 1:200 dilution and incubated at 4°C for two hours. 50 ul of protein A beads (Miltenyi Biotec) were added to the supernatant and incubated at 4°C for an additional hour. Samples were loaded on MACS separation columns (Miltenyi Biotec), followed by three washes with wash buffer (150 mM Tris, pH 7.4, 125 mM NaCl, and 1% NP-40). Protein complexes were eluted in 30 uL of pre-warmed 1X sample buffer.

#### Human TRIM5α co-immunoprecipitation

Sub-confluent HEK293 cells grown in 10-cm dishes were transfected with 24 μg total plasmid DNA using Lipofectamine 2000 (Invitrogen), following the manufacturer's protocol. Forty-two hours post-transfection, cells were washed with 5 ml ice-cold PBS and lysed with 800 μl ice-cold lysis buffer ((50 mM Tris, pH 7.5, 150 mM NaCl, 1% Triton X-100, 1 mM EDTA 10% glycerol), supplemented with protease inhibitor cocktail (Roche). Crude cell lysates were collected, transferred to pre-chilled 2 ml microcentrifuge tubes, and centrifuged at 10,000 × *g *for 10 minutes. The clarified lysate was transferred to pre-chilled microcentrifuge tubes. To prepare antibody conjugated beads, 2 μg of antibody (mouse anti-flag M2; Sigma F1804) was conjugated to 50 μL of Protein G Dynabeads^® ^(Invitrogen) following the manufacturer's protocol. The beads were washed three times with 1 ml ice-cold lysis buffer, resuspended in 50 μL lysis buffer, and added to the clarified cell lysates. After two hours rotating at 4°C, the bead-immune complexes were washed five times with 1 ml ice-cold lysis buffer, resuspended in 100 μL of 1 × Laemmli sample buffer, incubated at 100°C for five min.

### Western Blotting

Samples were loaded into a 10% polyacrylamide gel for SDS-polyacrylamide gel electrophoresis (SDS-PAGE). After separation, the proteins were transferred to nitrocellulose membrane (Bio-Rad) and detected by incubation with anti-HA conjugated to Horseradish Peroxidase (HRP) (Roche), anti-FLAG (Sigma) and anti-Myc. Secondary antibodies conjugated to HRP (Thermo Scientific) were used where necessary and antibody complexes were detected using SuperSignal™ West Femto Chemilluminescent Substrate (Thermo Scientific). Chemiluminescence was detected using the UVP EC3™ Imaging System (UVP LLC).

### Immunofluorescence

HeLa cells stably expressing YFP-rhTRIM5α were allowed to adhere to fibronectin-treated glass coverslips and fixed with 3.7% formaldehyde (Polysciences) in 0.1 M PIPES, pH 6.8 [piperazine-*N*, *N*'-bis(2-ethanesulfonic acid)] (Sigma). Monoclonal and polyclonal primary antibodies raised against various proteasomal subunits were used (Enzo Life Sciences, Table 1). Primary antibodies were secondarily labeled with Cy5 fluorophore-conjugated donkey anti-mouse or anti-rabbit antibody (Jackson ImmunoResearch). Images were collected with a DeltaVision microscope (Applied Precision) equipped with a digital camera (CoolSNAP HQ; Photometrics), using a 1.4-numerical aperture 100× objective lens, and were deconvolved with SoftWoRx deconvolution software (Applied Precision).

#### Image Analysis

20 Z-stack images were acquired using identical acquisition parameters. Surfaces for cytoplasmic bodies in all samples analyzed were defined by using a fluorescence threshold (600 relative fluorescence units) for YFP-rhTRIM5α, and all YFP-rhTRIM5α bodies over a volume of 0.011 μm^3 ^were used in the analysis. Deconvolved images were analyzed for PSMC2, RPT5, Alpha 4, Alpha 6, and 20S mean fluorescence intensity (MFI) in cytoplasmic bodies using the Surface Finder function of the Imaris software package (Bitplane) and the data was plotted in Prism (Graphpad Software Inc) for statistical analysis.

### Forster resonance energy transfer (FRET)

#### Immunofluorescent acceptor photobleaching in fixed cells

Cells stably expressing HA-rhTRIM5α [39] were seeded on coverslips at a subconfluent density. Coverslips were fixed with 3.7% formaldehyde (Polysciences) in 0.1 M PIPES [piperazine-*N*, *N*'-bis(2-ethanesulfonic acid)], pH 6.8. Cells were immunostained with a rabbit anti-HA primary antibody (Sigma) and mouse anti-PSMC2 or rabbit anti-20S primary antibodies (Enzo Life Sciences). Primary anti-HA antibody was secondarily labeled with Cy5-conjugated anti-rabbit antibody (Jackson ImmunoResearch), and proteasomal subunits were secondarily labeled with anti-mouse or anti-rabbit Alexa546 (Invitrogen). Cy5 fluorophore was bleached for total of two minutes every five seconds while fluorescence intensities were detected in the Alexa546 and Cy5 channels. Using SoftWoRx software, maximum intensities were analyzed over the course of the experiment for Alexa546 and Cy5 and graphed in Microsoft Excel.

#### Fluorescent protein acceptor photobleaching in live cells

FRET by acceptor photobleaching was performed as previously described [24]. Progressive acceptor photobleaching was performed as follows: 50 images were obtained at 10-second intervals for both donor (CFP: excitation 427/10, emission 473/30, 100 ms exposure) and acceptor (YFP: excitation 504/12, emission 542/27, 40 ms exposure), with a period of acceptor photobleaching (excitation 504/12) between each acquisition. The CFP/YFP fluorescence intensity of each cell in the field was quantified in Metamorph, and FRET efficiency was calculated from the CFP initial and final fluorescence values, according to E = 1-(F_prebleach_/F_postbleach_).

Fluorescence imaging was performed with an inverted microscope equipped with a 1.49 numerical aperture objective, and a back-thinned CCD camera (iXon 887; Andor Technology, Belfast, Northern Ireland). Image acquisition and acceptor photobleaching was automated with custom software macros in Meta-Morph (Molecular Devices Corp., Downingtown, PA) that controlled motorized excitation/emission filter wheels (Sutter Instrument Co., Novato, CA) with filters for CFP/YFP/mCherry (Semrock, Rochester NY). The progressive photobleaching protocol was as follows: 100-ms acquisition of CFP image and 40-ms acquisition of YFP image, followed by 10-s exposure to YFP-selective photobleaching (504/12-nm excitation).

#### E-FRET in live cells

E-FRET was performed as previously described [44].

E-FRET was calculated according to:

$$\text{E}=\frac{{\text{I}}_{\text{DA}}-a\left({\text{I}}_{\text{AA}}\right)-d\left({\text{I}}_{\text{DD}}\right)}{{\text{I}}_{\text{DA}}-a\left({\text{I}}_{\text{AA}}\right)+\left(\text{G}-d\right)\left({\text{I}}_{\text{DD}}\right)}$$

where I_DD _is the intensity of fluorescence emission detected in the donor channel (472/30 nm) with 427/10 nm excitation; I_AA _is the intensity of fluorescence emission detected in the acceptor channel with 542/27 nm emission and 504/12 nm excitation; I_DA _is the intensity of fluorescence emission detected in the "FRET" channel with 542/27 nm emission and 427/10 nm excitation; and *a *and *d *are cross-talk coefficients determined from acceptor-only or donor-only samples, respectively. We obtained a d value of 0.894 for CFP and a value of 0.108 for YFP. G is the ratio of the sensitized emission to the corresponding amount of donor recovery, which was 3.2.

## Abbreviations

rhTRIM5α: Rhesus macaque TRIM5α; RT: Reverse transcription; FRET: Forster resonance energy transfer.

## Competing interests

The authors declare that they have no competing interests.

## Authors' contributions

ZL designed, performed experiments, and wrote the manuscript. SH designed and performed experiments. SS designed and performed experiments. JR performed experiments. JS performed experiments. SLR designed experiments. JL designed experiments. EMC designed experiments and wrote the manuscript. All authors read and approved the final manuscript.

## Supplementary Material

Additional file 1 **Subcellular localization and characterization of proteasomal subunit antibodies using immunoflurescence**. HeLa cells seeded on glass coverslips were fixed with 3.7% Formaldehyde (Methanol Free) in PIPES buffer (pH 6.8). Following fixation they were stained with various antibodies to proteasome subunits and imaged using a DeltaVision deconvolution microscope. Click here for file

Additional file 2 **TRIM5α associates with proteasome subunit PSMC2 and 20S core particle in cells during immunofluorescence based FRET**. (A) HeLa cells stably expressing HA-rhTRIM5α were stained for HA using a primary anti-HA antibody, followed by a secondary antibody conjugated to Cy5 and endogenous PSMC2 using a mouse monoclonal antibody, followed by a secondary antibody conjugated to Alexa546. Z-stack images were collected and individual channel images were used to create the merged panels. (B) HeLa cells stably expressing HA-rhTRIM5α were stained for HA using an antibody conjugated to Cy5 and endogenous 20S core particle using a mouse monoclonal antibody conjugated to Alexa546. Z-stack images were collected and individual channel images were used to create the merged panels. Click here for file

Additional file 3 **YFP signal is progressively bleached during photoacceptor FRET**. (A) HeLa cells were transfected with CFP-rhTRIM5α and YFP-PSMC2. Images were collected throughout the acquisition process. Here we show CFP and YFP signal at the beginning (left) and at the end of the acquisition process (right). (B) HeLa cells were transfected with CFP-rhTRIM5α and YFP empty vector. Images were collected throughout the acquisition process. Here we show CFP and YFP signal at the beginning (left) and at the end (right) of the acquisition process. Click here for file
