# Supplementary figures and images for: TRIM5α associates with proteasomal subunits in cells while in complex with HIV-1 virions
Source: Retrovirology. 2011 Nov 12;8:93. doi: 10.1186/1742-4690-8-93 (PMC3279310; doi:10.1186/1742-4690-8-93)

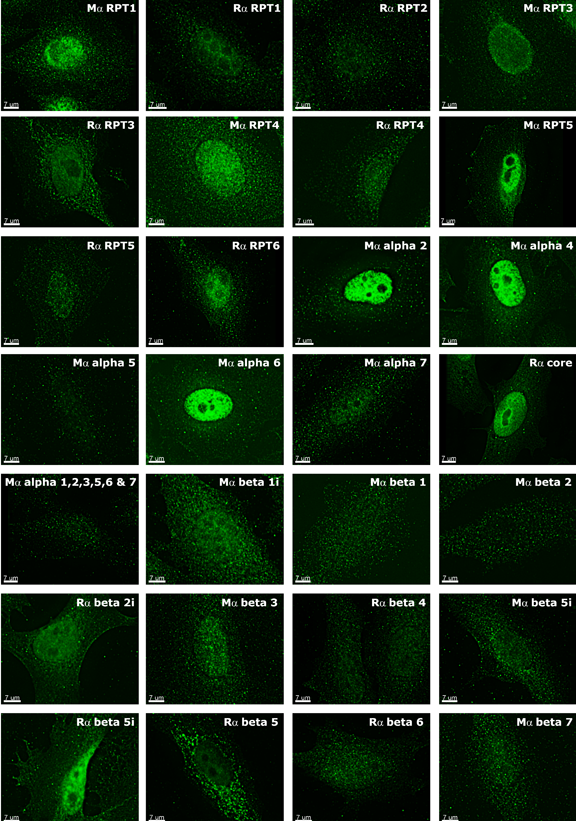

Supplement: Additional file 1 — Subcellular localization and characterization of proteasomal subunit antibodies using immunoflurescence. HeLa cells seeded on glass coverslips were fixed with 3.7% Formaldehyde (Methanol Free) in PIPES buffer (pH 6.8). Following fixation they were stained with various antibodies to proteasome subunits and imaged using a DeltaVision deconvolution microscope. [file 1742-4690-8-93-S3.TIFF]

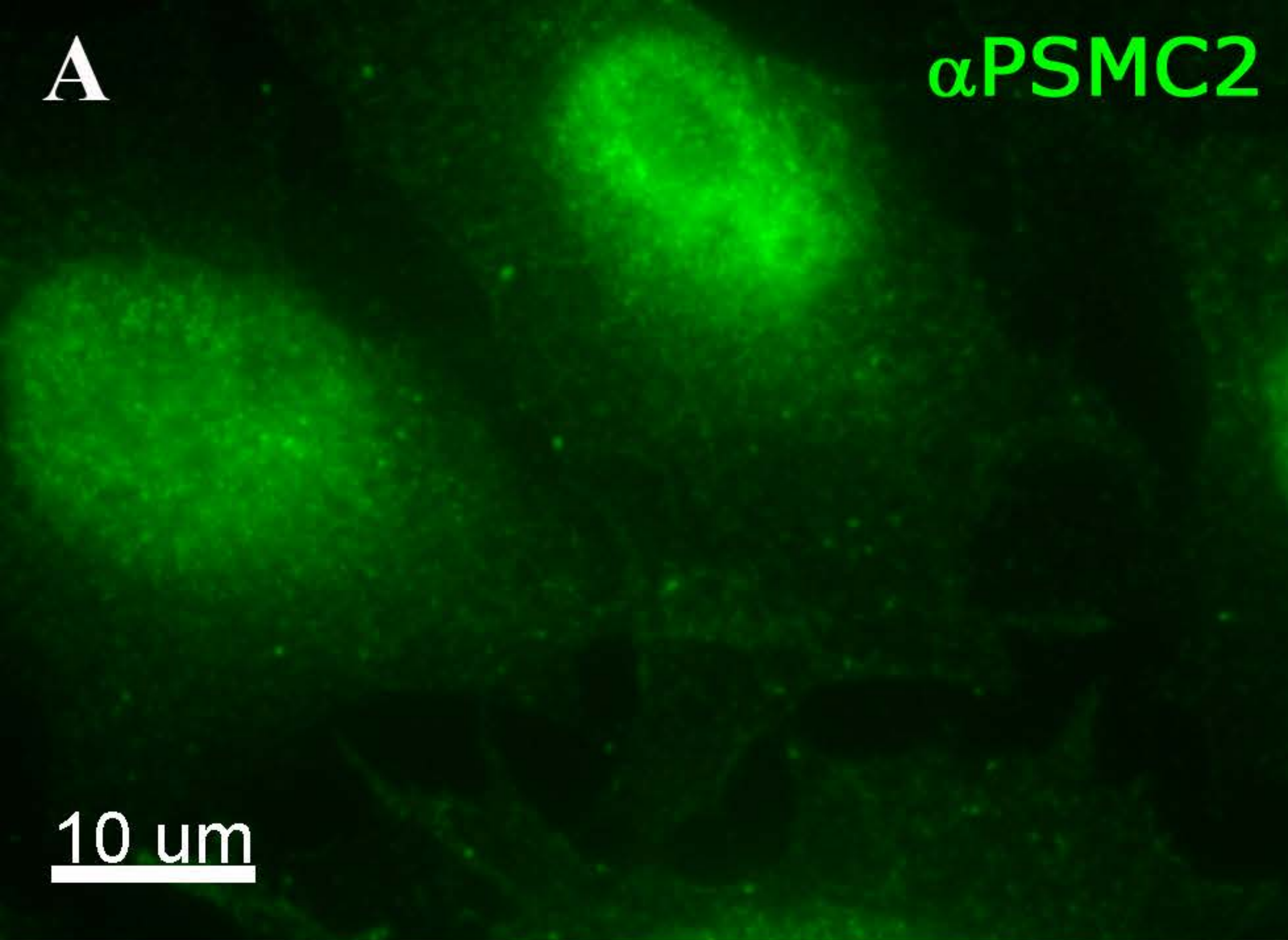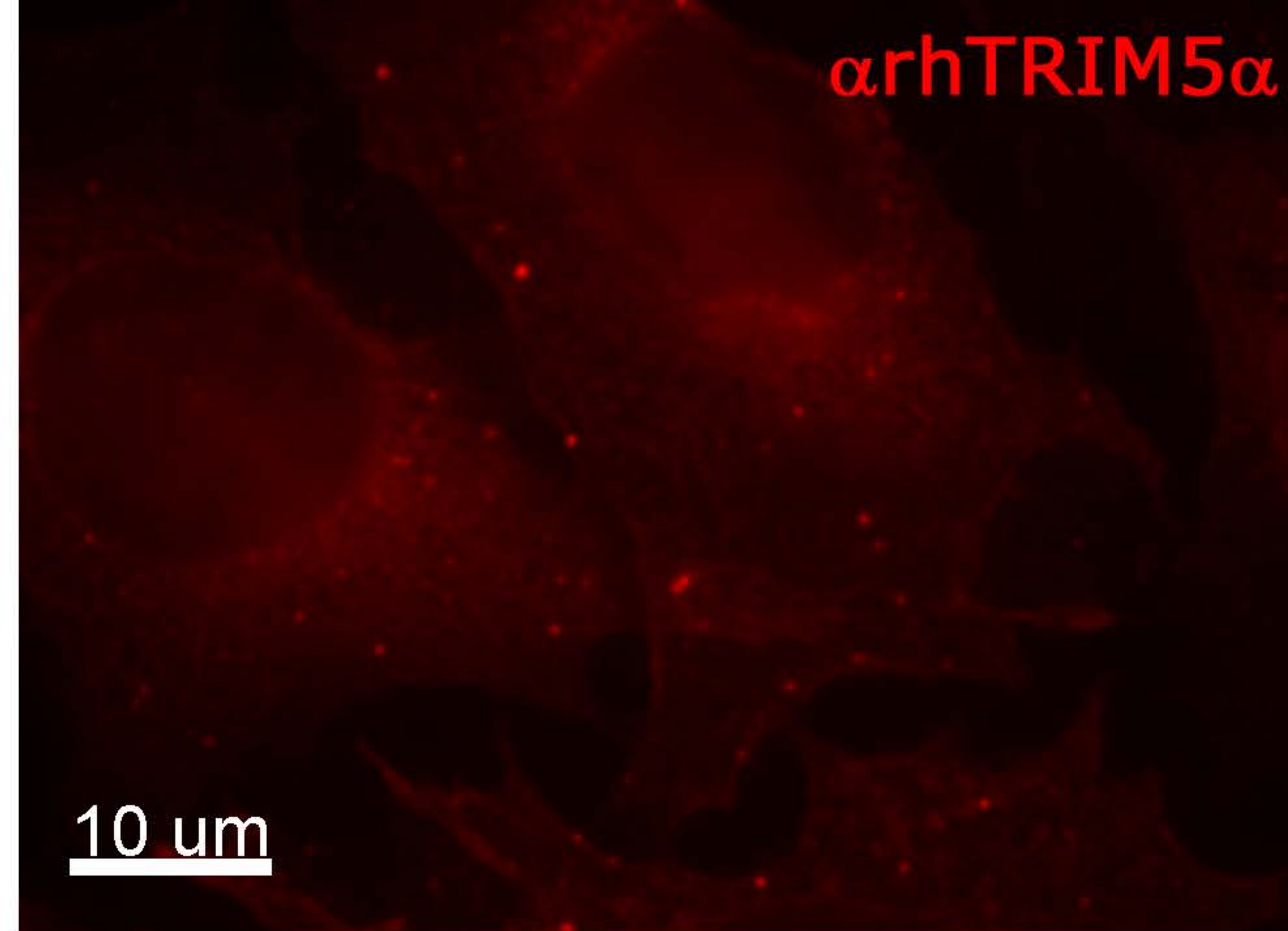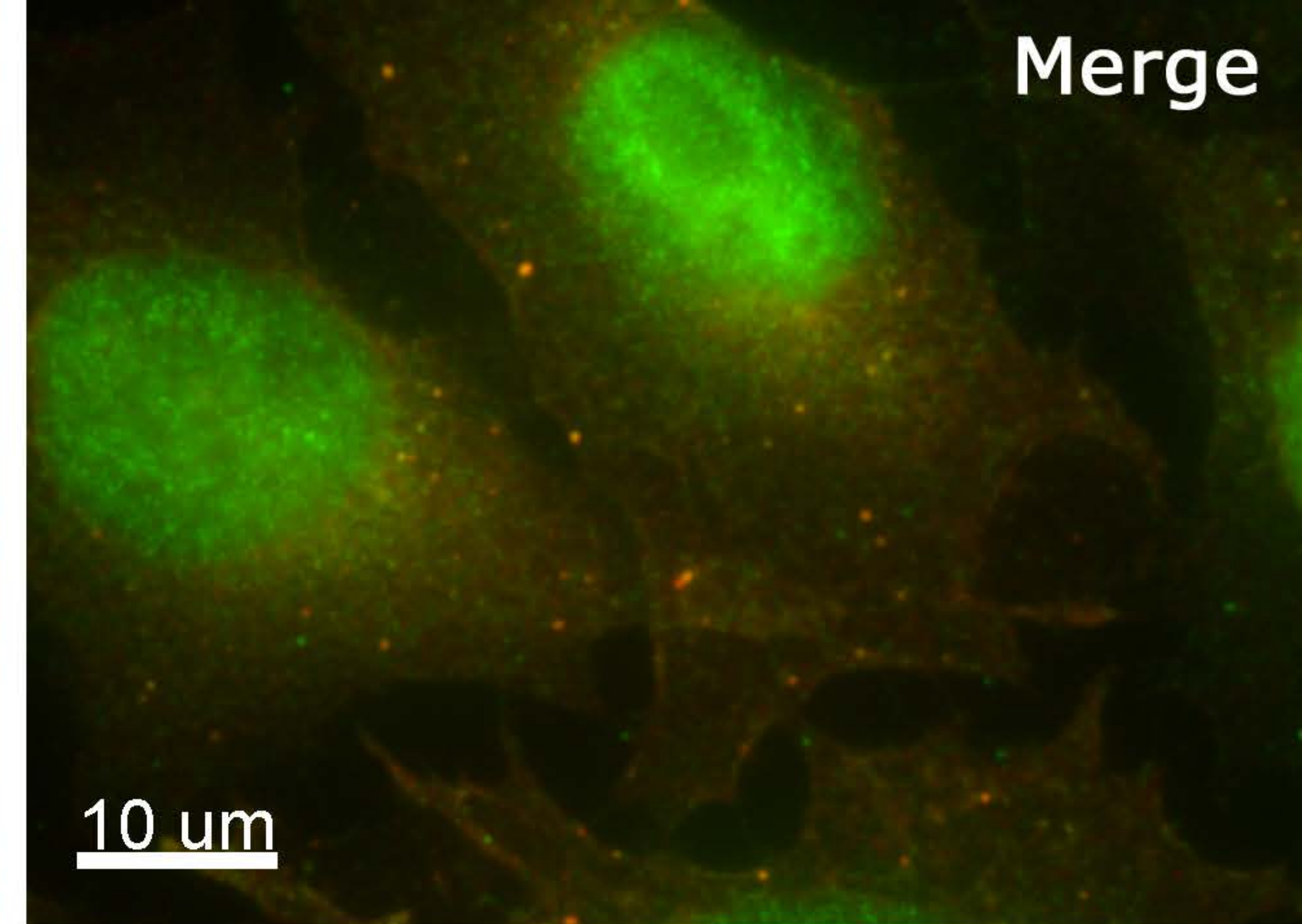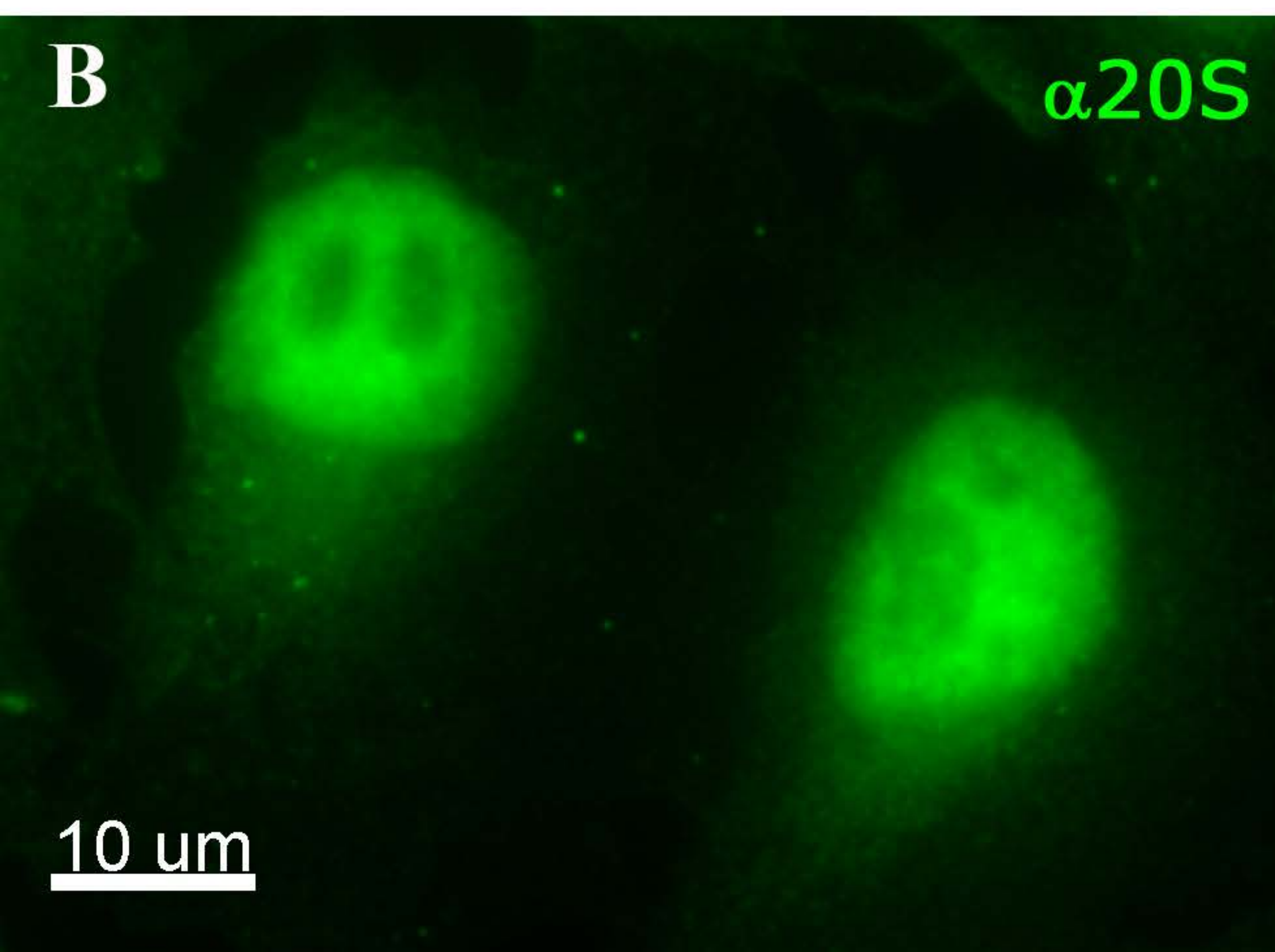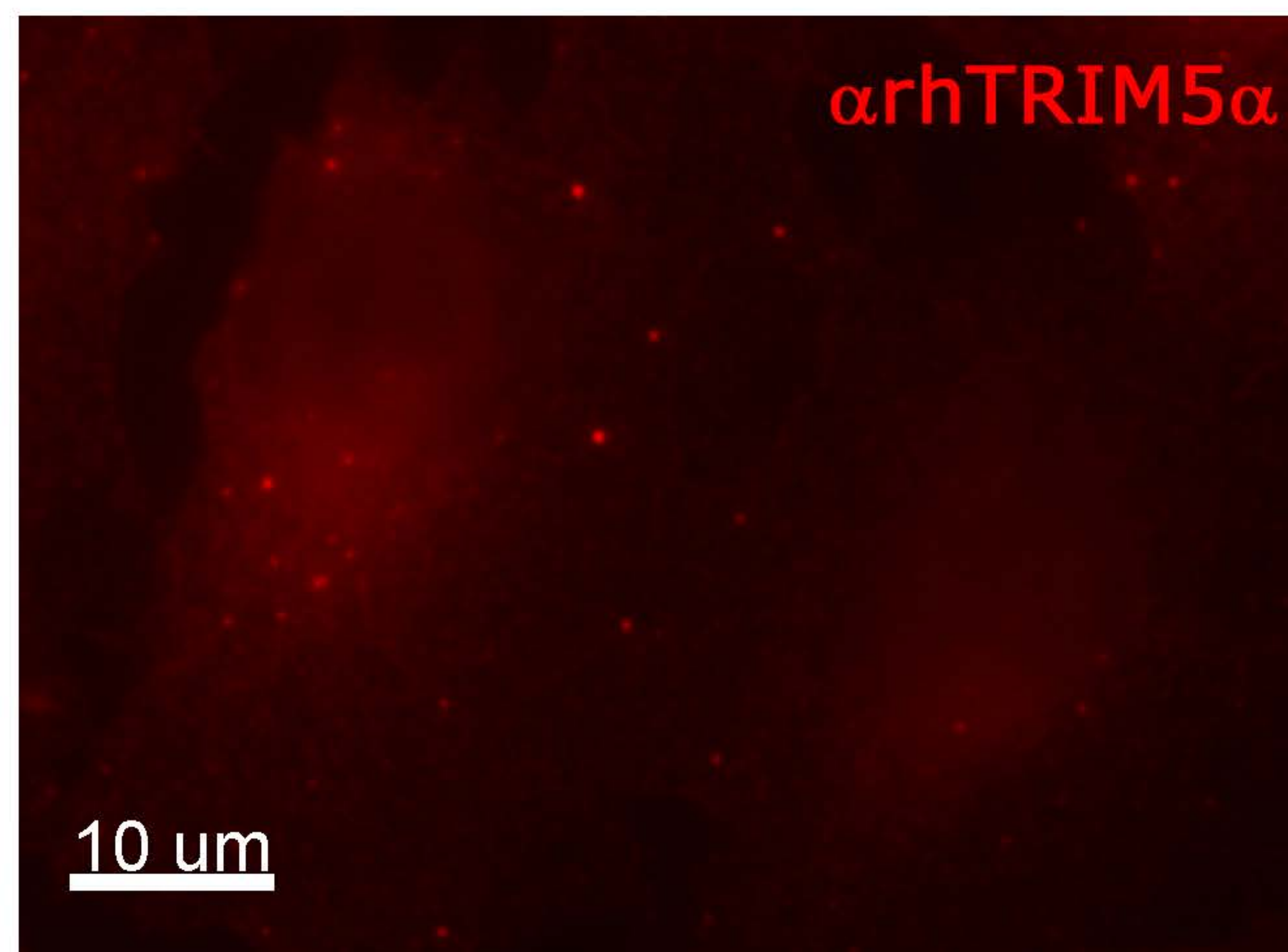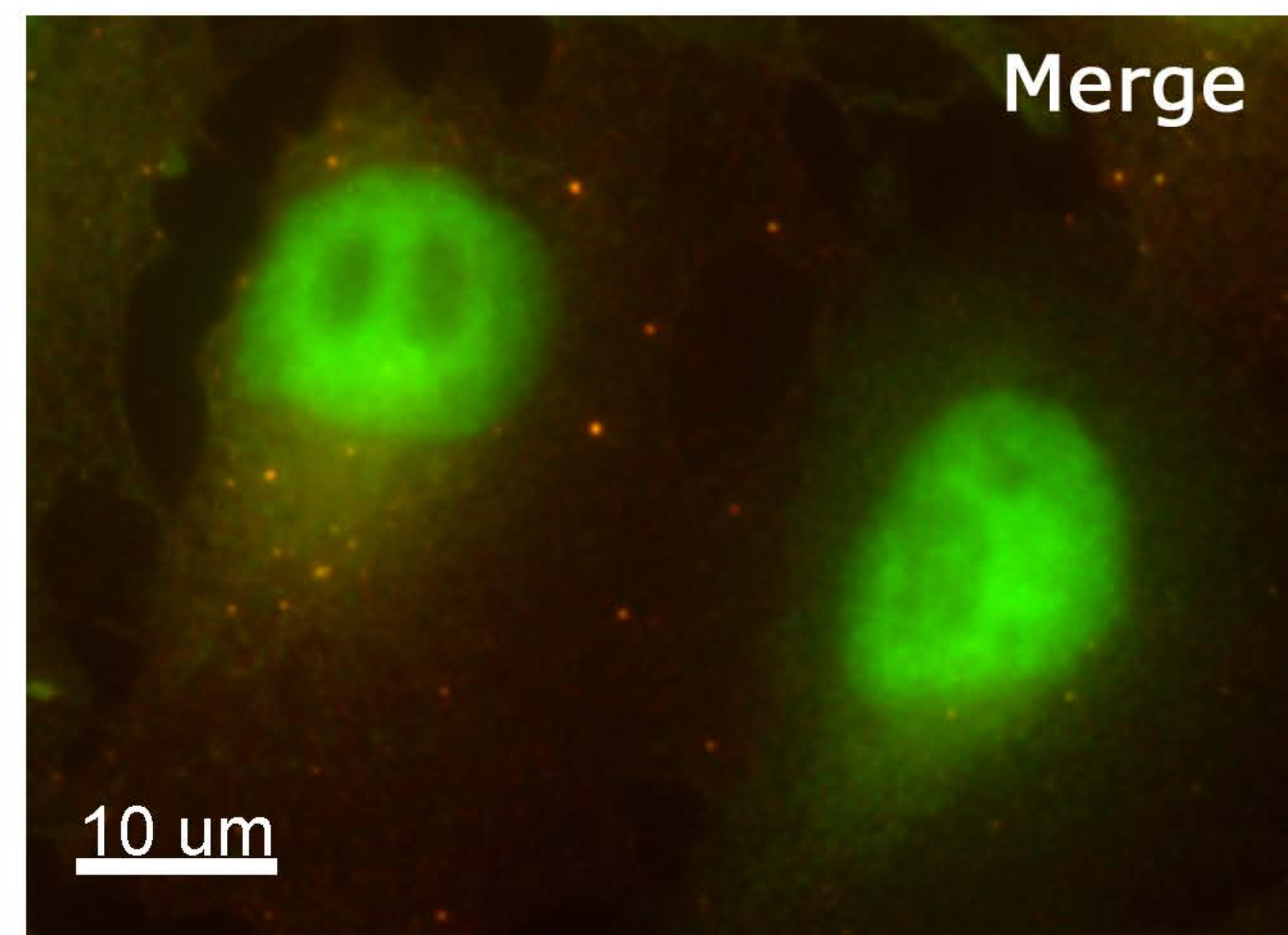

Supplement: Additional file 2 — TRIM5α associates with proteasome subunit PSMC2 and 20S core particle in cells during immunofluorescence based FRET. (A) HeLa cells stably expressing HA-rhTRIM5α were stained for HA using a primary anti-HA antibody, followed by a secondary antibody conjugated to Cy5 and endogenous PSMC2 using a mouse monoclonal antibody, followed by a secondary antibody conjugated to Alexa546. Z-stack images were collected and individual channel images were used to create the merged panels. (B) HeLa cells stably expressing HA-rhTRIM5α were stained for HA using an antibody conjugated to Cy5 and endogenous 20S core particle using a mouse monoclonal antibody conjugated to Alexa546. Z-stack images were collected and individual channel images were used to create the merged panels. [file 1742-4690-8-93-S1.PDF]

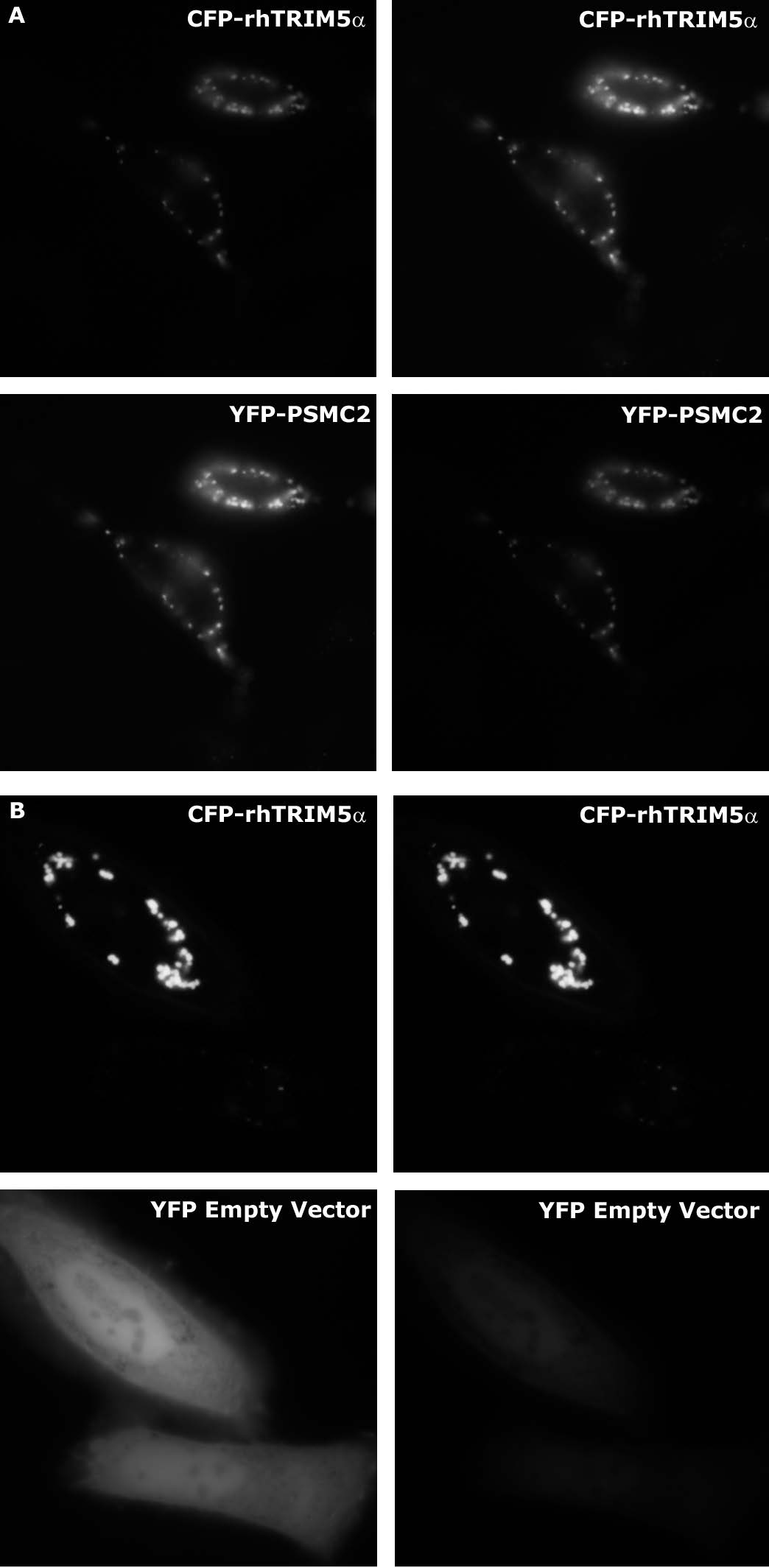

Supplement: Additional file 3 — YFP signal is progressively bleached during photoacceptor FRET. (A) HeLa cells were transfected with CFP-rhTRIM5α and YFP-PSMC2. Images were collected throughout the acquisition process. Here we show CFP and YFP signal at the beginning (left) and at the end of the acquisition process (right). (B) HeLa cells were transfected with CFP-rhTRIM5α and YFP empty vector. Images were collected throughout the acquisition process. Here we show CFP and YFP signal at the beginning (left) and at the end (right) of the acquisition process. [file 1742-4690-8-93-S2.TIFF]
